# Report of the Nitrous Oxide Committee

**Published:** 1873-01

**Authors:** 


### ARTICLE VII.

### *Report of the Nitrous Oxide Committee.*

The second and final report of the Joint Committee of the Odontological Society and the Dental Hospital staff, constituted to investigate the action of nitrous gas, was presented to the meeting of the former body, held on Monday evening last.

The report was read by Mr. Harrison, the chairman of the Committee, who explained that the delay in its presentation was due to the difficulty of following out the investigation except at the expense of much time.

The points submitted to the Committee for investigation, he remarked, were (1)the manner in which the agent acted as an anæsthetic, (2) the mode of treatment necessary in the event of the occurrence of alarming symptoms from the use of the gas, (3) the possibility and best mode of prolonging the anæsthetic effect of the gas, (4) the best mode of preparing and storing the gas, (5) the discovery of any anomalous effects during its exhibition, and (6) the mode of meeting these effects.

The Committee found it impossible to undertake *en masse* special experiments in regard to the first point, and this duty was delegated to Dr. Frankland, Mr. Coleman, and Mr. Braine. The former, as the result of his investigations, came to the conclusion that the nitrous oxide gas when inhaled underwent very little if any change. Rabbits were made to live for a certain time in atmospheres of nitrous oxide of known composition after the lungs had been emptied as far as possible of the residual air or filled with oxygen, and the atmosphere was subsequently analysed at different intervals of time after inhaling the nitrous oxide atmosphere, the result being as stated. Mr. Coleman's experiments on the human subject gave similar results; but Mr. Braine had not yet made his report. The general conclusion to be drawn from the experiments was that nitrous oxide induced anæsthesia by preventing oxidation.

In regard to the second point, the Committee stated that in the case of alarming symptoms coming on, artificial respiration should be resorted to at once, care being taken to draw the tongue well forward.

With regard to the third point—viz., the possibility of prolonging the anæsthetic effect, the Committee stated that this could be effected in many ways; in the case of operations on the mouth, by continuing to give the gas through the nose by a nose-piece, or by jetting the gas into the mouth at each inspiration, the nose being closed by a spring clip. In the case of operations upon parts other than the mouth, the inhalation of the gas could be kept up from time to time. The Committee expressed their regret that, inasmuch as anæsthesia could be kept for several minutes, the gas was not used in place of chloroform more frequently in the many minor operations of surgery, and they wished to direct the attention of surgeons to this point.

As to the fourth part, many well-known means had been recently introduced.

In regard to the fifth and sixth points, the Committee were glad to say that with the improvement in the making and administration of the gas, the occurrence of anomalous symptoms, excitement, hysteria, fainting, &c., had become less and less frequent. These conclusions were based upon 58,000 administrations.

As to the mode of death, it was certain that the respiration stops in fatal cases in dogs before the heart ceases to beat; the gas acted upon the nervous centres controlling the respiratory act; hence the value of artificial respiration and electricity should death be impending.

The Committee in their report next dealt with the mode and circumstances under which the nitrous oxide may be used with other anæsthetics, showing that the former does not interfere with the latter; and they conclude by pointing out what a safe anæsthetic is the nitrous oxide gas when used with ordinary care.—*London Lancet.*

